# Red Ginseng as an Ergogenic Aid: A Systematic Review of Clinical Trials

**DOI:** 10.20463/jenb.2016.0034

**Published:** 2016-12-31

**Authors:** Nan Hee Lee, Hyun Chul Jung, Sukho Lee

**Affiliations:** 1 Department of Counseling, Health, and Kinesiology, College of Education and Human Development, Texas A&M University-San Antonio, San Antonio USA; 2 Department of Kinesiology, College of Health and Pharmaceutical Sciences, University of Louisiana Monroe, Monroe USA

**Keywords:** Red Ginseng, Aerobic Capacity, Anaerobic Capacity, Antioxidant Function, Fatigue Recovery

## Abstract

**[Purpose]:**

This systematic review was performed to summarize clinical trials assessing the effect of Red Ginseng (RG) supplementation on exercise performance and fatigue recovery.

**[Methods]:**

Two English databases (PUBMED, MEDLINE) and two Korean databases (KISS, RISS) were used as systematic searching engines. We included only articles written in the English and Korean languages. Clinical trials, which evaluated exercise performance and recovery variables with RG supplementation, were included in this review. The methodological quality of all studies was assessed using the Cochrane Risk of Bias tool. Analysis was conducted with Comprehensive Meta-Analysis Version 3.

**[Results]:**

In total, 135 potentially relevant studies were identified, and 14 studies were included. Overall, the aerobic capacity (VO_2max_, heart rate, time to exhaustion, shuttle run, and anaerobic threshold) exhibited no improvement with RG supplementation. In anaerobic capacity (peak power, mean power, and 30 m dash run), no significant improvements with RG supplementation was described in most of the studies. The antioxidant function predominantly measured by levels of superoxide dismutase (SOD) and malondialdehyde (MDA) showed mixed results. Red Ginseng’s effects on fatigue recovery were evaluated using lactate as a main outcome. Two studies observed significant effects while other 5 studies showed no significant effects.

**[Conclusion]:**

The clinical effects of RG have been assessed in various conditions. Although the number of studies presented in this review is small and results of studies are mixed, it is hypothesized that this review article may provide useful guideline to design and conduct future studies investigating efficacy of RG supplementation on exercise performance and fatigue recovery in human trials.

## INTRODUCTION

An ergogenic aid can be defined as an equipment, technique, and substance used for enhancing sports performance. Ergogenic aids can be classified into different forms such as nutritional, pharmacological, and physiological aids. They range from use of accepted techniques such as high altitude training to consumption of illegal substances such as steroid. The efficacy of many of these is controversial, whereas the side effects are evident. An herb is a plant or plant part used for its scent, flavor, or therapeutic properties. In general, taking herb medication is considered as a safe practice. There are different forms of herbs such as tablets, capsules, powders, and fresh leaf. People use herbal medicines to maintain or improve their health. Herbs have been used to improve performance (both endurance and strength), facilitate recovery from work and exercise, maintain health, build muscle mass, and reduce body fat^1^-^3^. Ginseng is one of the most widely used herbs for human physical and mental performance, which includes several species and is prepared by various methods^4^.

The term ‘ginseng’ usually refers to the species Panax ginseng, known as Korean ginseng or Chinese ginseng. Ginseng root extracts have long been used in traditional oriental medicine to restore energy and enhance well-being^1^. Ginseng is a perennial herb which is indigenous to Korea, China (Panax ginseng C.A. Meyer), Himalaya (Panax pseudo-ginseng), Vietnam (e.g. Panax vietnamensis), Japan (e.g. Panax japonicus), and North America (Panax quinquefolium). Ginseng is available in many forms: whole root, root powder (white ginseng), steamed root powder (red ginseng), teas, tinctures, and standardized root extracts containing known and reproducible amounts of ginsenosides. The recognized primary active components of ginseng are a group of 30 different triterpene saponins, also referred to as ginsenosides, which vary in content and relative proportions among different species of ginseng^5^^,^^6^.

In exercise and sports science, ginseng is believed to be a physical performance enhancer^1^. Chen et al (2012)^1^ reported that Panax ginseng supplements may enhance physical and mental performance if taken over a long enough period of time and in sufficient doses. Panax ginseng, when administered at an adequate dosage (between 200 and 400 g/day) for a period of longer than 8 weeks may improve physical performance^1^. In general, ginseng supplements are safe, although individual variability exists and potentiation with stimulants may occur^7^^,^^8^.

Cultivated ginseng can be classified into four types: 1) fresh ginseng, 2) white ginseng, 3) red ginseng (steamed and dried process), and 4) wild ginseng^9^^,^^10^. RG contains bioactive compounds such as ginsenosides and phenolic compounds, and the compounds can vary according to the conditions of the heat process^11^. Extracts of red and white ginseng contain different ginsenosides^12^. Previous studies demonstrated that RG contain a large dose of ginsenosides and saponin through specific manufacturing processes such as steaming and drying ginseng^13^. Together with ginsenoside Rg3, a nitrogen-containing component and ginsenoside 20(R)-Rh1 were detected as characteristic components of RG, whereas malonyl ginsenoside Rb1/ isomer and malonyl ginsenoside Rg1/isomer were found to be characteristic components of WG^14^.

In particular, Korean Red Ginseng has ginsenoside such as Rg3, Rg 5, Rh1, and Rk1^15^^,^^16^. For these reasons, the identification of bioactive components in RG has been regarded as an objective of scientific study^17^^,^^18^. Recently, several studies have reported that RG has biological activities, such as vasodilation, antihypertension, antioxidant, anti-inflammatory, and improving cognitive function^19^-^22^.

RG study for exercise performance has been conducted predominantly with respect to aerobic capacity. RG supplementation demonstrated increase in the concentration of hemoglobin in the blood^23^. It also showed stimulation of mitochondrial metabolism in the muscles^24^. RG has been reported to improve the blood and organ lipid profile when combined with exercise^13^. It contains various biological and psychological activities and may also alleviate fatigue-related disorders. RG supplementation showed positive effect on fatigue resistance capacity, glycogen sparing, and oxidation of free fatty acid during prolonged sub maximal exercise^25^.

In contrast, effect of RG supplementation on anaerobic capacity does not look promising as compared to aerobic capacity^26^. A study by Engels, et al (2001)^27^ reported no significant changes in anaerobic capacity after 8 weeks of RG supplementation. Similarly, Yoon, et al (2001)^28^ also showed that there were no improvements in maximal and mean powers in Wingate power test. However, Hwang, et al (2004)^29^ reported that RG supplementation can reduce generation of oxidative free radical resulting in possible positive effect on exercise performance.

However, the number of studies and quality of RG studies are very limited as compared to the studies investigating regular ginseng. Also, there are only a few studies written in the English language available to scientific community.

Therefore, we conducted systematic review to summarize the current evidence of RG supplementation on exercise performance and fatigue recovery both written in the English and Korean languages. We anticipate that this review will provide valuable information about current ongoing research on RG to our colleagues and help them to design and conduct future research projects.

## METHOD

### Eligibility of studies

The inclusion criteria in this study were as follows: 1) RG as sole supplementation, 2) Studies involving a control or a placebo group, 3) Evaluated acute and chronic effects of RG supplementation, 4) Oral administration of RG in the form of capsules, powders or liquids, and 5) Assessed the efficacy of RG treatment on exercise performance and fatigue recovery. On the other hands 1) RG supplementation combined with other products, 2) Nonoral administration of compounds, and 3) animal studies, case studies, and uncontrolled trials were excluded in this study.

### Data Sources

The following four electronic English and Korean databases were searched with restriction of language (English and Korean) from their respective inceptions up to June 2016: the Korean Studies Information Service System (KISS), Korea Institute of Science and Technology Information, Research Information Service System (RISS), US National Library of Medicine National Institutes of Health (PUBMED), and Advancing the Health of Healthcare (MEDLINE). The search terms used were ‘‘Red Ginseng’’ and ‘‘exercise’’ in the English and Korean languages. Unpublished articles were excluded. The references in all located articles were also searched. Hard copies of all articles were obtained. Figure 1 illustrates process of study selection.

### Types of interventions

Trials that included extract of RG, regardless of age, gender, or dose, were included. According to the processing status, we excluded studies targeted on animals. We compared placebo or no treatment to RG supplementation treatments on exercise performance, antioxidant function, and fatigue recovery.

### Data collection

The data (author, publication year, country, sample size, conditions of the participants, intervention, dosage, treatment duration, outcome measures, main results, and language) were extracted by a standard form. All the articles were read by two independent reviewers, who extracted data from the articles according to predefined criterion.

### Assessment of risk of bias

The risk of bias for each study was evaluated by the ‘Risk of Bias’ assessment with Review manager 5.1 (Copenhagen: The Nordic Cochrane Centre, The Cochrane Collaboration, 2011) in this study. The following sources were assessed: 1) sequences generation, 2) allocation concealment, 3) subjects and personnel blinding, 4) assessor blinding, 5) incomplete outcome data, and 6) selective outcome report. Our review used unclear (U), low (L) and high (H) as keys for judgments. Differences in opinions between the reviewers were settled through discussion.

**Table 1.** Summary of clinical of red ginseng for aerobic and anaerobic capacity, antioxidant, and fatigue recovery function

No	Author (year)	Gender (M/F/Both)	Condition	Group (n)	Dose per day (duration, forms)	Main outcomes	(Std diff in Means, 95% CI, p-value) Main Results	Language
1	Choi & Lee (2008)^33^	Nr	Healthy individual	RG: 7 CG: 7	200 ml (12 wk, liquid)	1) VO_2max_	1) 0.369 [-0.687, 1.426], P = 0.493	Korea
						2) HR_max_	2) -0.352 [-1.408, 0.704], P = 0.513	
						3) VE	3) 0.368 [-0.688, 1.425], P = 0.494	
						4) Lactate	4) -0.327 [-1.382, 0.727], P = 0.543	
2	Choi (2008)^34^	Nr	Healthy individual	RG: 8 CG: 7	200 ml (12 wk, liquid)	1) VO_2max_	1) 0.458 [-0.570, 1.486], P = 0.382	Korean
3	Jung et al. (1993)^35^	M	Healthy individual	RG: 7 PG: 7	1,500 mg (12 wk, powder)	1) Lactate	1) -0.081 [-1.129, 0.967], p=0.880	Korean
4	Kim et al. (1996)^36^	M	Healthy individual	RG: 6 PG: 6	3 g (8 wk, capsule)	1) HR_recovery_	1) 0.090 [-1.042, 1.222], P = 0.876	Korean
						2) Lactate	2) 2.139 [0.720, 3.558], P =0.003	
						3) LDH	3) -0.696 [-1.862, 0.469], P = 0.242	
5	Kim et al. (2006)^37^	M	Healthy individual	RG: 10 PG: 10	900 mg (12 wk, capsule)	1) SOD	1) 1.957 [0.891, 3.023], p=0.000	Korean
						2) GPX	2) 1.712 [0.687, 2.736], P = 0.001	
						3) MDA	3) -2.207 [-3.318, -1.095], P = 0.000	
6	Kim (2012)^38^	M	Healthy individual	RG: 8 CG: 8	200 ml (one time, liquid)	1) HR_rest_	1)-0.055 [-1.035, 0.926], P = 0.913	Korean
						2) Lactate	2) -0.076 [-1.056, 0.905], P = 0.879	
7	Kim et al. (2016)^39^	M	Healthy individual	RG: 11 PG: 11	5 g (one time, Nr)	1) Peak power	1) 0.028 [-0.808, 0.864], P = 0.949	English
						2) Mean power	2) 0.283 [-0.557, 1.123], P = 0.509	
						3) Lactate	3) -1.209 [-2.118, -0.300], P = 0.009	
						4) LDH	4) -0.360 [-1.202, 0.483], P = 0.403	
8	Lee & Kim (1999)^40^	F	Healthy individual	RG: 7 PG: 7	3,000 mg (8 wk, capsule)	1) SOD	1) 1.565 [0.368, 2.763], p=0.010	Korean
						2) MDA	2) -1.040 [-2.156, 0.076], p = 0.068	
						3) CAT	3) 1.122 [-0.005, 2.249], p = 0.051	
9	No & Park (2013)^41^	M	Healthy individual	ERG: 6 EG: 6	100 mg (24 wk, liquid)	1) VO_2Max_	1) 0.035 [-1.096, 1.167], P = 0.951	Korean
						2) AT	2) 0.762 [-0.410, 1.934], P = 0.202	
						3) Time to exhaustion	3) 0.406 [-0.734, 1.549], P = 0.486	
						4) RBC	4) 0.629 [-0.530, 1.788], P = 0.288	
						5) Hemoglobin	5) 2.034 [0.640, 3.427], P = 0.004	
						6) Hematocrit	6) 0.891 [-0.295, 2.077], P = 0.141	
10	Park et al. (2000)^42^	M	Diabetic patient	ERG: 6 EG: 6	3.0 g (12 wk, capsule)	1) VO_2Max_	1) 0.239 [-0.896, 1.375], P = 0.680	Korean
						2) HR_Max_	2) -0.031 [-1.163, 1.100], P = 0.957	
						3) VE	3) 0.031 [-1.100, 1.163], P = 0.957	
11	Park & Kim (2004)^43^	M	Diabetic patient	ERG: 6 EG: 6	3.0 g (12 wk, capsule)	1) SOD	1) 1.065 [-0.144, 2.275], p=0.084	Korean
						2) CAT	2) 1.260 [0.021, 2.498], P = 0.046	
						3) MDA	3) -0.193 [-1.327, 0.941], P = 0.739	
12	Park & Choi (2012)^44^	Both	Healthy individual	RG: 10 PG: 10	320 mg (8 wk, capsule)	1) HR_rest_	1) -0.657 [-1.577, 0.243], P = 0.152	Korean
						2) Shuttle run	2) 2.467 [1.304, 3.630], P = 0.000	
						3) 30m dash run	3) -2.654 [-3.856, -1.452], P = 0.000	
						4) SOD	4) 0.118 [-0.759, 0.995], P = 0.792	
						5) MDA	5) -0.665 [-1.566, 0.235], P = 0.148	
						6) Lactate	6) -0.140 [-1.018, 0.737], P = 0.754	
13	Yoon et al. (2008)^45^	M	Healthy individual	ERG: 7 EPG: 7	100 ml (12 wk, liquid)	1) VO_2Max_	1) 0.068 [-0.980, 1.116], P = 0.899	Korean
						2) Recovery VO_2_	2) 0.302 [-0.752, 1.355], P = 0.575	
						3) Mean power	3) 0.167 [-0.883, 1.216], P = 0.756	
						4) Peak power	4) 0.609 [-0.463, 1.681], P = 0.265	
14	Yoon et al. (2012)^46^	Nr	Healthy individual	RG: 12 PG: 10	100 ml (one time, liquid)	1) Time to exhaustion	1) 0.343 [-0.502, 1.189], P = 0.426	Korean
						2) Peak power	2) 0.032 [-0.807, 0.807], P = 0.940	
		Nr	Healthy individual	RG: 10 PG: 10	100 ml (6 wk, liquid)	1) VO_2Max_	1) 0.573 [-0.322, 1.467], P = 0.210	Korean
						2) Time to exhaustion	2) 0.075 [-0.801, 0.952], P = 0.866	
						3) Peak power	3) 0.306 [-0.576, 1.188], P = 0.496	
						4) Mean poser	4) 0.061 [-0.816, 0.938], P =0.891	
						5) Lactate	5) -0.055 [-0.932, 0.822], P = 0.902	

M: male; F: female; Nr: not reported; RG: red ginseng group; CG: control group; PG: Placebo group; EG: exercise group; ERG: exercise with red ginseng group; EPG: exercise with placebo group; VE: ventilation; SOD: superoxide dismutase; MDA: malondialdehyde; GPX: glutathione peroxidase; CAT: catalase; LDH: lactate dehydrogenase.

### Analysis

The studies were organized according to their primary outcomes. Estimated effect size for each outcome of included studies was calculated by comparative analysis with each control intervention individually. The outcomes were presented as mean difference (MD) and 95% Confidence Interval (CI). Analysis was conducted with Comprehensive Meta Analysis (CMA) 3.0 version. All statistical analyses were independently cross-checked by a statistician.

## RESULTS

### Study Selection and Description

We considered 135 articles screened from 4,283 potentially relevant articles (Figure 1). After assessing full-text articles for eligibility, a total of 82 articles were evaluated. Subsequently, 68 were excluded because they used animal models (16 articles), did not meet the eligibility criteria (18 articles), or because of other reasons (31 articles: irrelevant articles with our research topic). The three studies excluded for intervention conditions were as follows: a mixture of RG plus peonia radix (PR)^30^, while two studies employed vitamin E or electrolyte^31^^,^^32^.

**Figure 1.**
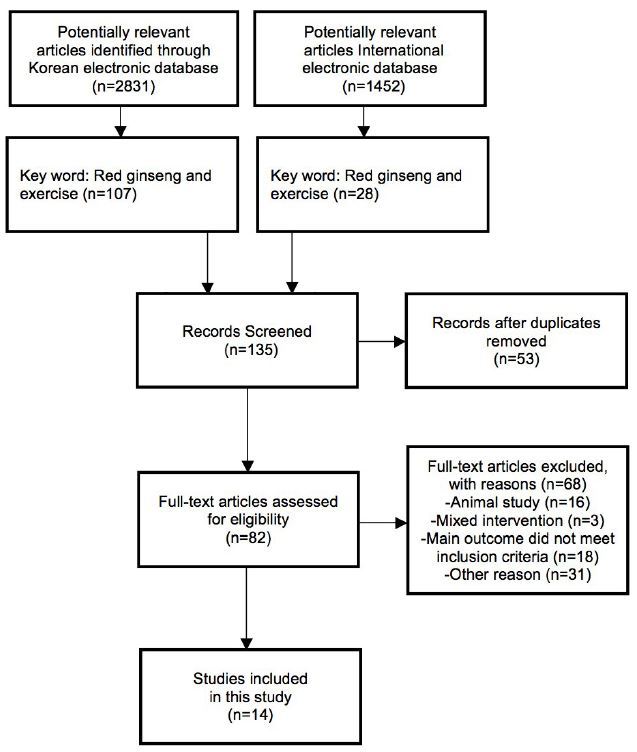
Process of study selection

Finally, 14 studies met our inclusion criteria ^33^-^46^. Thirteen studies^33^-^38^^,^^40^-^46^ were published in the Korean language, and one study^39^ was written in the English language.

Two studies were described as ‘randomized’; one study was specified as ‘double-blind’, two studies were specified as ‘single-blind’, and thirteen studies did not state blinding. Thirteen studies were designed as parallel, and one study was cross-over.

### Intervention

The trials used RG as an intervention for exercise performance, antioxidant, and fatigue recovery after intense exercise. Eight studies compared the efficacy of RG with placebo^35^-^37^^,^^39^^,^^40^^,^^44^-^46^ and six studies compared with control^33^^,^^34^^,^^38^^,^^41^^-^^43^. In two studies, the participants were characterized by diabetes^42^^,^^43^. Other twelve studies involved healthy individuals^33^-^41^^,^^44^-^46^. Three different supplementation forms were used across the studies, capsules^36^^,^^37^^,^^40^^,^^42^^-^^44^, liquids^33^^,^^34^^,^^41^^,^^45^^,^^46^^,^ and powder^35^ and one study did not report the details39. In terms of the variables, nine studies employed aerobic capacity^33^^,^^34^^,^^36^^,^^39^^,^^41^^,^^42^^,^^44^-^46^, four studies investigated anaerobic capacity^37^^,^^40^^,^^43^^,^^44^, four studies investigated antioxidants^37^^,^^40^^,^^43^^,^^44^^,^ and seven studies investigated fatigue recovery^33^^,^^35^^,^^36^^,^^38^^,^^39^^,^^44^^,^^46^. RG doses ranged from 900 mg to 5 g/day for capsules, 100 to 200 ml/day for liquid, and 1500 mg/day for powder.

### Risk of bias

Risks of bias from all 14 studies are summarized in Figure 2. Four studies^35^^,^^37^^,^^38^^,^^45^ used methods of random sequence generation. The risk of bias in sequence generation was considered as high risk in one study^36^. However, none of the studies reported the methods of randomization such as computerized. Most of the studies did not clearly report the manner in which allocation concealment was generated, while five studies^35^-^38^^,^^45^ employed allocation concealment. One study^44^ was conducted with double blinded design (participants/researcher), while other two studies^40^^,^^41^ just reported ‘blinding (participants)’ design. None of the studies mentioned ‘adopted assessor blinding’. The risk of bias in terms of the incomplete outcome data and the selective reporting were unclear in the present review.

**Figure 1.**
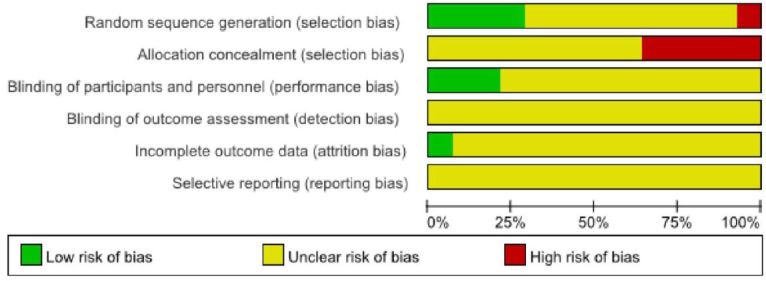
Process of study selection

### Effects of intervention

#### Aerobic and anaerobic capacity

Aerobic and anaerobic capacity were assessed in 9 ^33^^,^^34^^,^^36^^,^^38^^,^^41^^,^^42^^,^^44^-^46^ and 4 ^39^^,^^44^-^46^ studies, respectively. These studies measured 5 different variables (VO_2max_, heart rate; HR, time to exhaustion, shuttle run, and anaerobic threshold; AT), and terms of anaerobic capacity was also tested by 3 different variables (peak power, mean power, and 30 m dash run).

In case of aerobic capacity, VO_2max_ was the main variable evaluated by 6 studies, with HR measured in 5 studies, and time to exhaustion measured in 3 studies. Other variables like shuttle run test, AT, and biological variation (red blood cell; RBC, hemoglobin; Hb, and Hematocrit; Hct) were measured in one study each. A total 22 outcomes from 10 variables, all related to VO_2max_ outcomes^33^^,^^34^^,^^41^^,^^42^^,^^45^^,^^46^ showed no significant effects. The five HR outcomes^33^^,^^36^^,^^38^^,^^42^^,^^44^ also demonstrated no significant effects. Hemoglobin^41^ and shuttle run test^45^ showed positive significant effects. Other outcomes such as ventilation^33^^,^^42^^,^ RBC^41^^,^ HCT^41^^,^ recovery VO_2_^45^ AT^41^^,^ and time to exhaustion test^41^^,^^46^ were not significant.

In case of anaerobic capacity, peak power was the main outcome evaluated by 4 studies. Mean power outcomes were measured in 2 studies, and 30 m dash run was measured in one study. A total of 8 outcomes from 3 variables, most of the outcomes such as peak power^39^^,^^45^^,^^46^ and mean power^39^^,^^45^^,^^46^ showed no significant differences. In the study conducted by Park, et al. (2012), a significant difference on shuttle run test was observed^44^.

#### Antioxidant function

Four studies^37^^,^^40^^,^^43^^,^^44^ investigated the effects of RG related with intense exercise on antioxidant function measured by changes in levels of superoxide dismutase (SOD), malondialdehyde (MDA), catalase (CAT), and glutathione peroxidase (GPX). SOD and MDA were the main variables evaluated by all the studies associated with antioxidants^37^^,^^40^^,^^43^^,^^44^^,^ and CAT was measured in 2 studies^40^^,^^43^. Other variable, GPX was measured in one study^37^. A total of 11 outcomes were evaluated from 4 variables. In four SOD outcomes^37^^,^^40^^,^^43^^,^^44^^,^ two^37^^,^^40^ had positive effects and two^43^^,^^44^ were not significant. Most of the outcomes involving MDA^40^^,^^43^^,^^44^ were not significant excluding the study reported by Kim, et al (2006)^37^. In terms of CAT, one of those^43^ showed significant effect, while another study^40^ was not significant. In the study reported by Kim, et al. (2006) GPX showed significant mean differences^37^.

#### Fatigue recovery

The effect of RG on fatigue recovery was evaluated in 7 studies^33^^,^^35^^,^^36^^,^^38^^,^^39^^,^^44^^,^^46^. In all the studies related with fatigue variables, lactate was evaluated as a main outcome. In a total of 9 outcomes, 7 were lactate ^33^^,^^35^^,^^36^^,^^38^^,^^39^^,^^44^^,^^46^ and 2 were lactate dehydrogenase (LDH)^36^^,^^39^. Two outcomes^36^^,^^38^ on lactate observed significant effects, and other 5 outcomes^33^^,^^35^^,^^39^^,^^44^^,^^46^ were not significant. In terms of LDH outcomes^36^^,^^39^, no significant effects were observed.

## DISCUSSION

This study reviewed 14 published articles investigating the effect of RG on exercise performance and fatigue recovery in human trials. This review has differentiated significance compared to previous review articles in terms of summarizing only RG supplementation related with exercise. Furthermore, the present review represents a systematic evaluation of clinical trials published in the Korean and English literatures. Finally, 14 studies that met the inclusion criteria were categorized based on aerobic, anaerobic, antioxidant function, and fatigue recovery. We observed that previous studies primarily focused on aerobic capacity and fatigue recovery as variables of ergogenic ability. To the best of our knowledge, only 10 outcomes have shown that RG supplementation can help in exercise performance. Specifically, only 2 of 22 outcomes were in aerobic category, 1 of 8 outcomes was in anaerobic category, 5 of 11 outcomes were in antioxidant category, and 2 of 7 outcomes were in fatigue recovery category. VO_2max_ and HR were commonly evaluated as an aerobic capacity outcome. There was some evidence that RG may have an effect on aerobic capacity, with 2 of 22 outcomes partially demonstrating improvement in Hb, and shuttle run test score in RG groups compared with control groups. However, most of the studies showed that RG supplementation had no effects on aerobic outcomes including VO_2max_, HR, AT, VE, RBC, and time to exhaustion test. Most of the results clearly state that RG supplementation had no effects on anaerobic capacity and fatigue recovery. In terms of antioxidant function, 5 of 11 outcomes had positive significant effects. However, the outcomes observed heterogeneities between each variable, which means that the data in each category were inconsistent and statistically insufficient. Therefore, more studies are obliged to judge the effects clearly. Many studies conducted using animals have usually shown that RG may improve exercise performance; whereas, data from human subjects reveal contradictory results. We believe that the inference could be due to various methodological problems including small sample size, different dosage, and lack of placebo group. The actual composition of ginseng preparations has been a persistent issue, and differences in the effects of ginseng may be due to the dosage and type (powder, extract, etc) of RG consumed. There may be interactions with diet, lifestyle, exercise, and other drugs.

We wish to highlight some of the difficulties faced while conducting research on RG and offer some suggestions for future research. Most of the reported studies did not used standardized RG preparations. Moreover, the majority of studies did not describe a ginsenoside profile except for two articles^43^^,^^44^. None of the studies reported independent testing of the preparation to confirm purity, consistency, and stability. In addition, dose ranges varied among the studies (900 mg to 5 g/day for capsules, 100 to 200 ml/day for liquid, and 1500 mg/day for powder). Non-randomization may lead to a substantial overestimation of the effect size. It is also suggested that post-harvest handling processing for RG should be standardized using the identified characteristic components as chemical markers to ensure the quality and efficacy of RG.

In the methodological aspect, the quality of most of the studies was poor, as they did not clearly describe adequate randomization, blinding, and/or description of withdrawals and dropouts. In addition, none of the studies conducted statistical calculation to determine reasonable subject numbers. Moreover, we observed that interpretation of the results was incorrect based on statistical analysis. For instance, a study concluded that RG had effects on exercise performance despite no significant interaction by repeated measures ANOVA. Therefore, the limitations in methodological quality could induce the different interpretation of RG supplementation on exercise performance. As such, it is necessary to conduct further studies that are of high quality and with larger sample sizes to contribute towards formation of a definitive conclusion. Specifically, it is required to describe the accurate information of ginsenoside dosage, and ratios to evaluate the efficacy of RG as an ergogenic aid. In addition, future studies need to fulfill accurate research procedures.

This review may serve as a foundation for future systematic reviews and further studies, but it also has some limitations. First of all, data synthesis for this study was limited by heterogeneity between outcomes. Pooling results were confounding, which was inappropriate. This phenomenon may be caused by the small sample size and varied supplementation dosage. Another limitation was that this review article included studies written in the Korean and English languages.

## CONCLUSION

This review explores the scientific evidence for use of RG extracts as ergogenic aid for exercise performance. The results of this systematic review reveal that RG is not effective on aerobic and anaerobic capacity, antioxidant function, and fatigue recovery. However, we conclude that there is absence of strong scientific research evidence regarding the efficacy of RG on improving exercise performance in humans. In future studies, it is necessary to conduct further systematic review of high quality and involving large sample sizes with an aim to form a definitive conclusion.
